# Supplementary material for: Teaching computer architecture by designing and simulating processors from their bits and bytes
Source: PeerJ Comput Sci. 2024 Feb 19;10:e1818. doi: 10.7717/peerj-cs.1818 (PMC10909196; doi:10.7717/peerj-cs.1818)
Supplement: Supplemental Information 1 [file peerj-cs-10-1818-s001.pdf]

SUPPLEMENTARY

The supplementary material is divided into three distinct sections: **Section A Architecture Details** offers comprehensive information on the MuSe and DoMe architectures, encompassing details on the multiplication algorithm, ALU design, and data path control signals. In **Section B Simulator Details** we provide a detailed breakdown of the simulator structures through class descriptions. Lastly, **Section C Survey** delves into the specifics of the survey questions and the corresponding responses.

A ARCHITECTURE DETAILS

A.1 MuSe Architecture Design

In this section, we present detailed information on the multiplication algorithm, ALU design, data path, and control signals within the MuSe Architecture. The architecture's instructions, as outlined in Table 1, are further elaborated in Table 2, providing a comprehensive explanation for each instruction. Notably, the MuSe Architecture includes an instruction referred to as ***syscall***, which, while not mandatory for simulator functionality, serves the purpose of enabling programmers to display register contents on an LCD screen when invoked. It's worth mentioning that the ***jr*** and ***syscall*** instructions are exceptions within the architecture, as they do not make use of target and destination registers; however, they are categorized under the R-Format due to their utilization of source registers.

A.1.1 MuSe Architecture: The Multiplication Algorithm

Multiplication operations can be computationally intensive, often requiring significant processing time within the Arithmetic Logic Unit (ALU). In the MuSe Architecture, our approach to handling multiplication tasks follows the iterative algorithm proposed by Patterson and Hennessy, 2016. This methodology implements multiplication iteratively, utilizing registers and adders, as depicted in Fig. 1. To accommodate this iterative approach, the MuSe Architecture incorporates a specialized multiplication unit within the ALU. This unit comprises eight shift registers and eight 8-bit adders, tailored to efficiently handle multiplication operations.

A.1.2 MuSe Architecture: The ALU Design

The Arithmetic Logic Unit (ALU) serves as the central component of the CPU, responsible for executing all computational tasks. Within the MuSe Architecture, the ALU manages its operations through two input ports, referred to as input A and input B, along with an output port named output C. The ALU is highly versatile and capable of performing a wide array of calculations. The type of calculation to be executed is determined by the 3-bit ALU control lines denoted as F0, F1, and F2. These control lines are governed by the ALUOp control signal, which is introduced in the data path and changes dynamically as each incoming instruction is decoded.

Table 3 presents a comprehensive list of mathematical operations supported by the MuSe Architecture, along with their corresponding control line values. MuSe Architecture's ALU design accommodates eight distinct operations, each represented by specific combinations of these three control lines.

A.1.3 MuSe Architecture: The Data-path and Control Signals

The data-path within a processor serves as the central framework that interconnects essential hardware components, including functional units like the ALU, adders, memory, and registers. It is also equipped with a set of control signals that facilitate the coordination of different CPU units. For instance, when the load word (lw) instruction is executed, setting the memory read control signal to 1 enables reading data from memory. When designing the data-path for MuSe Architecture, the developers drew inspiration from the original MIPS data-path.

In the conventional MIPS architecture, there are eight distinct control signals (MIPS, 2001). However, in the design of MuSe Architecture, 11 control signals are integrated to accommodate various instructions effectively. These additional control signals, in addition to those inherited from traditional MIPS architecture, include the System Call (***syscall***), Jump Register (***JumReg***), and Shift Register (***Shift Reg***) control signals. Control signal values for each instruction in MuSe

Architecture are thoughtfully compiled in Table 4, aiming to facilitate and guide researchers in replicating the MuSe Architecture design.

A.2 DoMe Architecture Design

In this section, we delve into an in-depth exploration of the multiplication algorithm, ALU design, data path, and control signals incorporated within the DoMe Architecture.

The instructions utilized in the DoMe architecture are thoughtfully laid out in Table 2, accompanied by their usage in desktop simulators. Notably, it is important to highlight that the suffix "*-c*" is exclusive to the application in R-type instructions.

A.2.1 DoMe Architecture: The Multiplication Algorithm

In this section, we delve into the multiplication algorithm employed in the DoMe Architecture design phase. Following extensive research, the DoMe Architecture designers initially opted for the Wallace Tree multiplication method (Wallace, 1964). However, during the design phase presentation, their project co-advisor recommended an alternative multiplication approach. The advisor's advice was grounded in the realization that implementing an 8-bit Wallace Tree multiplier would entail significant costs and complexities, potentially posing challenges during the planned physical implementation phase. Consequently, the DoMe Architecture designers pivoted to adopt the multiplication algorithm suggested by the MuSe Architecture in **Section A.1.1 MuSe Architecture: The Multiplication Algorithm** to enhance feasibility and practicality.

A.2.2 DoMe Architecture: The ALU Design

DoMe Architecture has been meticulously crafted to provide support for a broader spectrum of instructions compared to the MuSe Architecture. Consequently, the repertoire of DoMe Architecture instructions includes division and exclusive or (***xor***) operations, in addition to the other eight operations. Recognizing the challenge of representing ten different operations with merely three control lines, the DoMe Architecture designers astutely incorporated an additional control line (F3). This design choice resulted in six unused control signals. Further elaboration on ALU operations and their corresponding control line values can be found in the supplementary materials. Furthermore, the ALU operation list for the DoMe Architecture, as delineated in Table 3, encompasses a more extensive array of operations and includes the ALU control lines (F3), a notable expansion in comparison to MuSe Architecture's ALU Design.

A.2.3 DoMe Architecture: The Data-path & Control Signals

In this section, we delve into the DoMe Architecture's distinctive approach to designing its data-path and control signals. As previously highlighted in Section 3.2.4, the traditional MIPS architecture encompasses eight distinct control signals. However, the MuSe Architecture augmented this count to 11. In contrast, the DoMe Architecture streamlined its control signals to a total of seven, achieved by the elimination of the Register Destination (RegDst) and Memory to Register (MemtoReg) control signals. An additional control signal, known as "jump," was introduced to facilitate the management of jump instructions. To accommodate DoMe architecture, all unit and control signal connections in the MIPS data-path were meticulously revised. Much like in the MuSe Architecture, the control signal values pertinent to DoMe Architecture can be found in Table 5.

B SIMULATOR DETAILS

B.1 MuSe Architecture Simulator

The architecture of the MuSe simulator is intricately composed of several well-defined classes, each dedicated to a specific aspect of the simulation. These classes encompass ALU, Controller Unit, Instructions, Instruction Memory, Data Memory, Register File, Program Counter, and Processor. Within the ALU class, the designers have favored the use of elementary operators for computational tasks rather than embarking on the intricate endeavor of implementing complex logic circuits such as full adders and multiplication logic. The Controller Unit class assumes the pivotal responsibility of assigning control signals to individual instructions through a meticulous evaluation of opcodes, ***is_jump*** flags, and ***is_imm*** values. The MuSe simulator primarily

revolves around three distinct instruction types, namely R-type, I-type, and J-type instructions. To streamline the management of shared attributes across these instruction types, the simulator extends these instructions from an abstract class named Instruction. The Instruction Memory is constructed as an array of instructions, initialized upon program commencement. Meanwhile, the Data Memory module houses a two-dimensional byte array augmented with a stack pointer, effectively governing all essential memory read and write operations. The Register File class boasts a roster of registers, encompassing eight pre-defined registers and proficiently manages both read and write operations in alignment with control signals. The Program Counter class is characterized by its simplicity, storing merely an integer value to represent the program counter value and adeptly governing its manipulation. The Processor class orchestrates the harmonious interaction of the previously mentioned classes, meticulously choreographing their workflow. The program commences with the initialization of instruction memory, and as it unfurls, the processor diligently fetches instructions in a loop. Notably, in the MuSe architecture, the absence of a pipeline implementation means that the simulator only progresses to the next instruction once the current instruction is successfully executed. The program culminates either when all instructions are executed or in the event of an encountered error.

B.2 DoMe Architecture Simulator

The intricate structure of the DoMe Simulator consists of an array of classes, with certain classes sharing functional similarities with their counterparts in the MuSe Simulator. The essential classes used in the DoMe Simulator include Registers, Instructions, Data Memory, Instruction Memory, Definitions, Instruction Functions, Assembler, and Processor. The Registers class, much like in the MuSe Simulator, serves as the repository for registers, with their initialization taking place here. The Instructions class stores the assembly instructions within an array and enables their retrieval by the Processor class through a systematic loop. Data Memory, much like its MuSe counterpart, plays a pivotal role in memory operations, with memory contents being stored within an array. It's noteworthy that, unlike the MuSe Simulator, the DoMe Simulator foregoes the inclusion of a dedicated ALU class. Instead, it features the Instruction Functions class, which defines a function for each instruction operation and establishes a linkage between function and instruction. This unique approach allows the simulator to directly access these functions and execute the requisite operations. The Assembler class within the DoMe Simulator shares similarities with the Control Unit class in the MuSe simulator, responsible for the assignment of control bits, opcodes, registers, and other essential values. The Definitions class serves as a valuable look-up table, pre-defining the properties of each instruction and is instrumental in aiding the Assembler class in the configuration of instruction attributes. Despite disparities in the implementation of specific components within their data-path, the designers of the DoMe Simulator are acutely aware that this aspect of the project places a distinct focus on augmenting the capacity and workflow of their architecture.

C SURVEY

This section delineates the survey questions and presents the responses provided by 50 students.

Question 1 aims to assess participants' perception of their hardware design knowledge and experience, both before and after the course (Fig. 2).

Question 2 measures the participants' self-reported self-learning ability before and after the course, which is a vital aspect of their educational growth (Fig. 3).

Question 3 gauges the change in participants' interest in working on low-level systems like Computer Hardware Engineering from before to after the course, helping understand how the course impacts their preferences (Fig. 4).

Question 4 investigates participants' awareness of developing software considering hardware limitations before the course, offering insights into their initial perspectives (Fig. 5).

Question 5 focuses on the impact of visual tools on the participants' learning experience throughout the course, examining how such tools influenced their engagement (Fig. 6).

Question 6 explores the influence of group work on the learning experience, uncovering the effect of collaborative learning on participants' perceptions (Fig. 7).

Question 7 assesses how hands-on experiences during the course influenced participants, providing insights into the practical aspects of their learning (Fig. 8).

Question 8 evaluates the effect of designing their own architecture on participants' learning experience, highlighting the significance of practical application in the course (Fig. 9).

Question 9 measures the impact of simulators and internet-shared tools on participants' learning experience, indicating the relevance of digital resources (Fig. 10).

Question 10 evaluates the overall satisfaction level of participants with the Computer Architecture course, summarizing their general contentment (Fig. 11).

Question 11 investigates the alignment between participants' expectations and the course's content and delivery, offering insights into how well the course met their initial anticipations (Fig. 12).

Question 12 assesses participants' confidence in their understanding of hardware design after completing the course, indicating the level of self-assuredness in their knowledge (Fig. 13).

Question 13 aims to measure how well participants believe the course prepared them for low-level system work like Computer Hardware Engineering (Fig. 14).

Question 14 captures participants' overall enjoyment of the course, providing a holistic perspective on their satisfaction and engagement with the educational experience (Fig. 15).

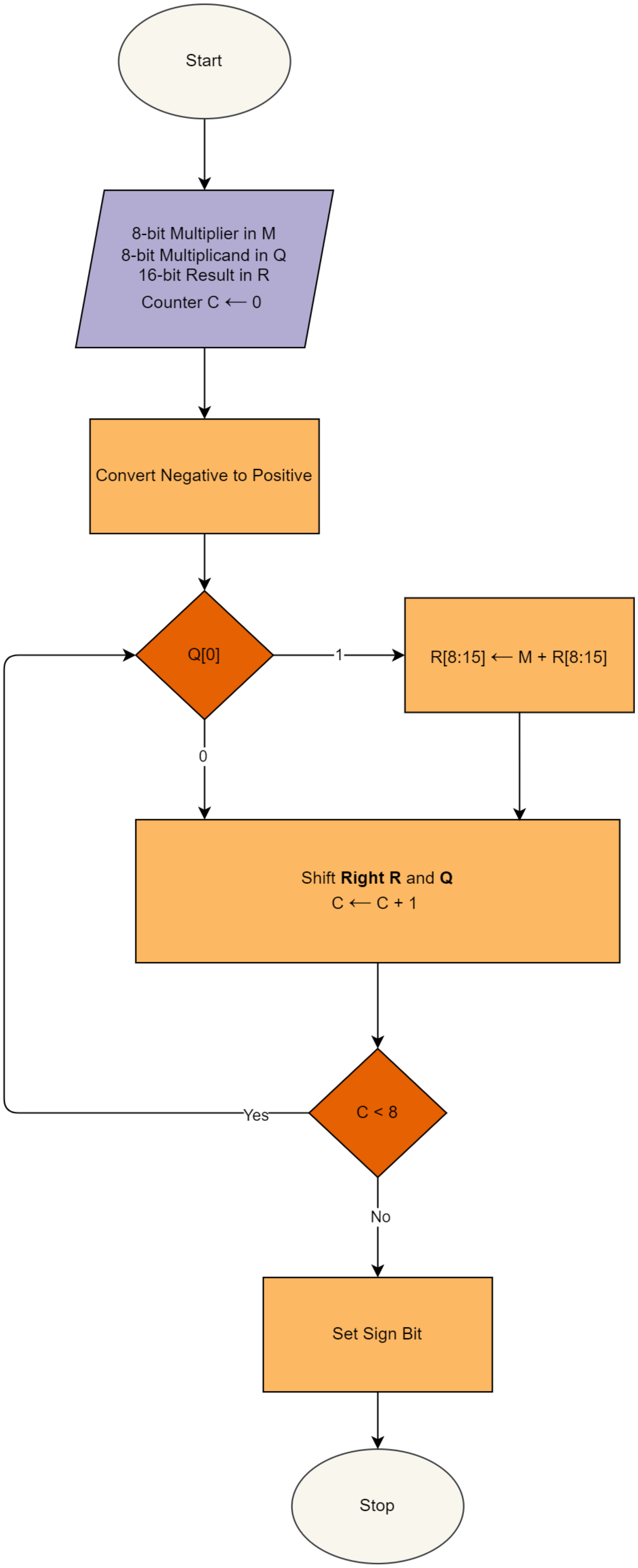

Figure 1. Multiplication Algorithm Flow Chart.

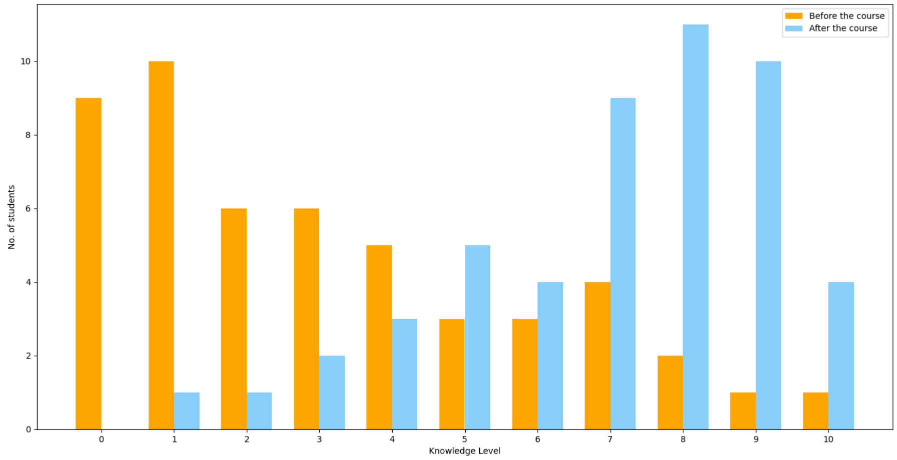

Figure 2. Results for *"How would you rate your knowledge and experience level about hardware design before/after the course?"*

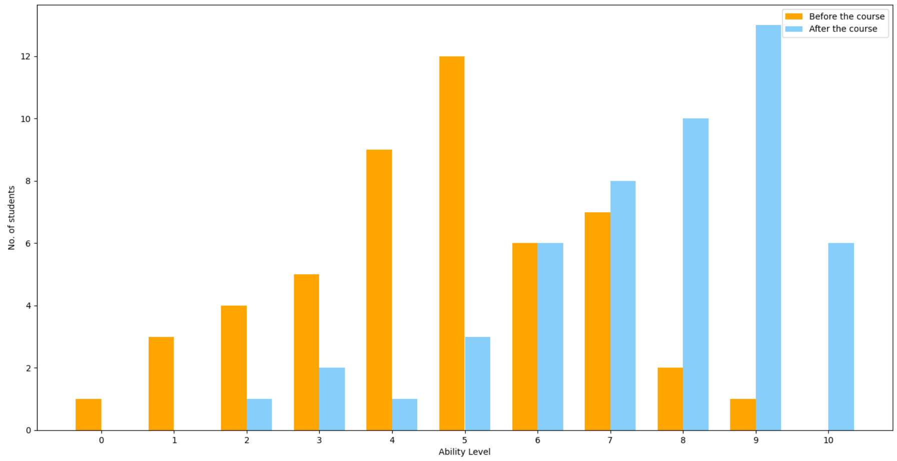

Figure 3. Results for *"How would you rate your self-learning ability before/after the course?"*

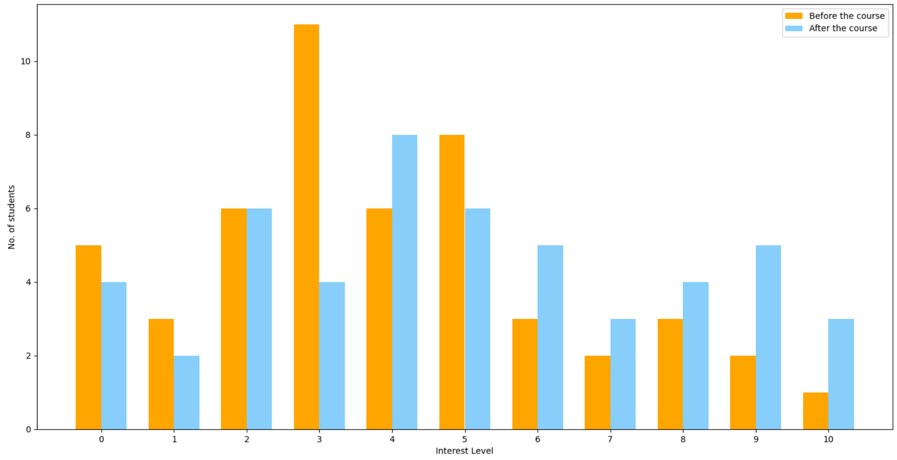

Figure 4. Results for *"How interested were you in working on low-level systems, such as Computer Hardware Engineering before/after the course?"*

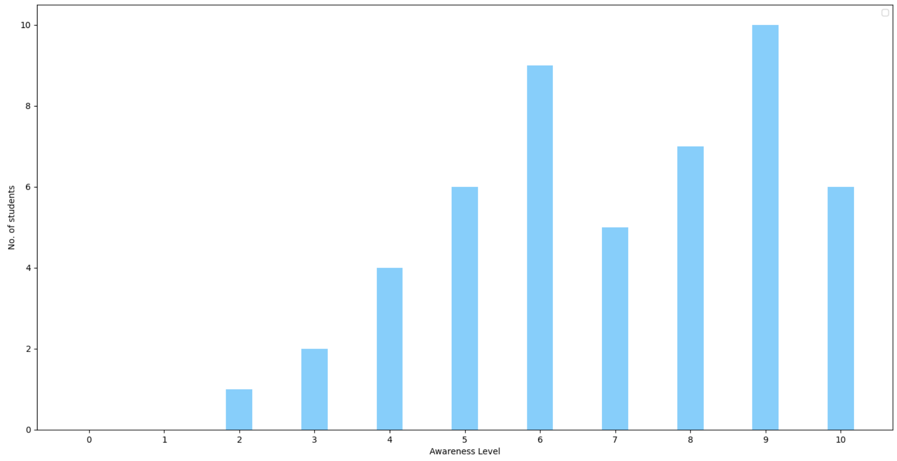

Figure 5. Results for *"How aware were you of developing software by considering hardware abilities and limitations before the course?"*

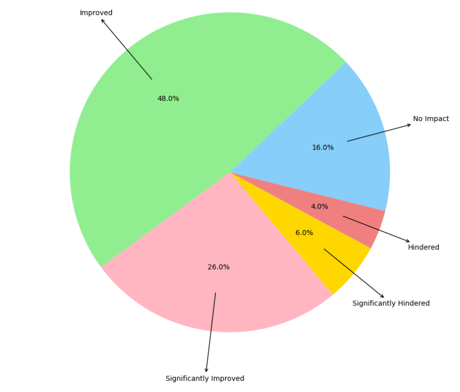

Figure 6. Results for ”*How did the use of visual tools during the course impact your learning experience?*”

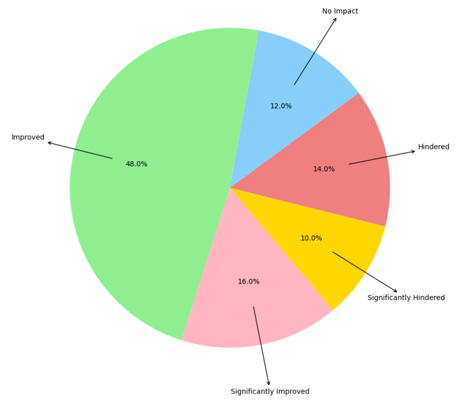

Figure 7. Results for ”*How did working in groups during the course affect your learning experience?*”

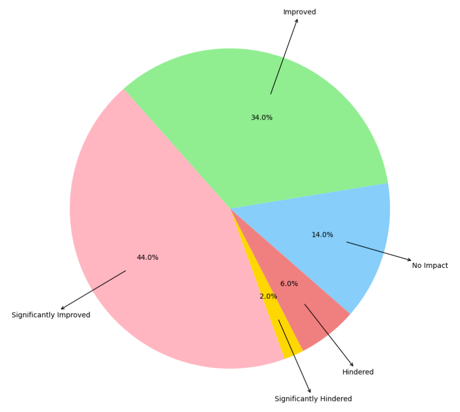

Figure 8. Results for ”*How did hands-on experiences during the course influence your learning experience?*”

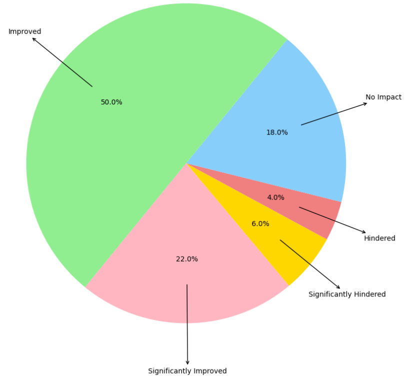

Figure 9. Results for *"How did implementing and designing your own architecture during the course affect your learning experience?"*

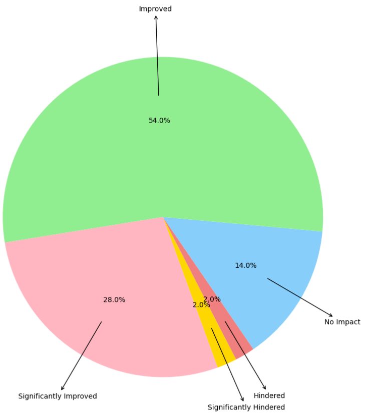

Figure 10. Results for *"How did using simulators and tools shared on the internet during the course impact your learning experience?"*

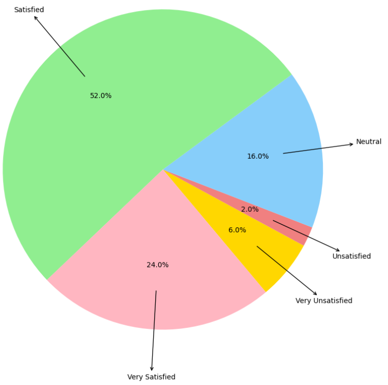

Figure 11. Results for *"How satisfied were you with the Computer Architecture course?"*

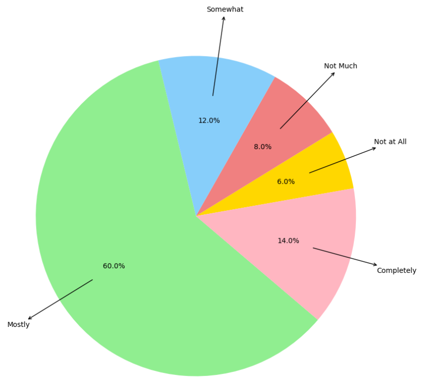

Figure 12. Results for *"To what extent did this course meet your expectations?"*

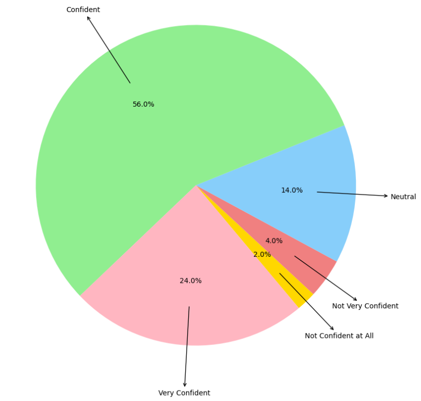

Figure 13. Results for *"How confident do you feel in your understanding of hardware design after completing this course?"*

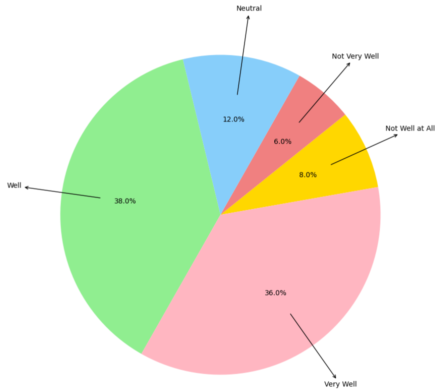

Figure 14. Results for *"How well do you think the course prepared you for low-level system work, such as Computer Hardware Engineering?"*

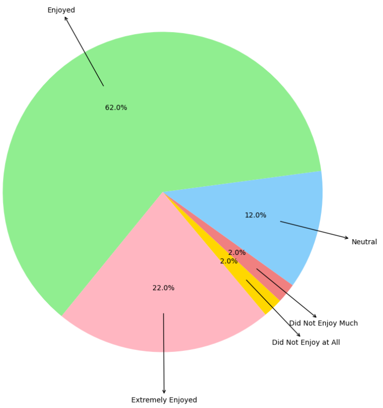

Figure 15. Results for *"How would you describe your overall enjoyment of this course?"*

Table 1. Instruction Format of each Architecture

MuSe Architecture	R-Type	Field	opcode	is_jump	is_imm	rs	rt	rd	unsued	Total
		Bit	3	1	1	3	3	3	2	16
	I-Type	Field	opcode	is_jump	is_imm	rs	rt	immediate		Total
		Bit	3	1	1	3	3	5		16
	J-Type	Field	opcode	is_jump	is_imm	label				Total
		Bit	3	1	1	11				16
DoMe Architecture	R-Type	Field	opcode	control_bit	rt	rs	function code			Total
		Bit	4	1	3	3	5			16
	I-Type	Field	opcode	control_bit	rt	immediate				Total
		Bit	4	1	3	8				16

Table 2. Instruction List (**Not a MUST instruction*)

MuSe Architecture	R-Type	000	0	0	rs	rt	rd	unused	ADD
		001	0	0	rs	rt	rd	unused	SUB
		100	0	0	rs	rt	rd	unused	MUL
		010	0	0	rs	rt	rd	unused	AND
		011	0	0	rs	rt	rd	unused	OR
		101	0	0	rs	rt	rd	unused	SLL
		110	0	0	rs	rt	rd	unused	SRL
		101	1	0	rs	rt	rd	unused	*SYSCALL
		111	0	0	rs	rt	rd	unused	SLT
		001	1	0	rs	rt	rd	unused	JR
	I-Type	101	0	1	rs	rt	imm[4:0]		LUI
		111	0	1	rs	rt	imm[4:0]		SLTI
		100	0	1	rs	rt	imm[4:0]		MULI
		001	0	1	rs	rt	imm[4:0]		BEQ
		011	0	1	rs	rt	imm[4:0]		BNE
		000	0	1	rs	rt	imm[4:0]		SW
		010	0	1	rs	rt	imm[4:0]		LW
	J-Type	000	1	0	imm[10:0]				JAL
		001	1	0	imm[10:0]				J
DoMe Architecture	R-Type	1000	control_bit	rt	rs	00010			AND(C)
		1000	control_bit	rt	rs	01000			OR(C)
		1000	control_bit	rt	rs	00000			ADD(C)
		1000	control_bit	rt	rs	01101			SUB(C)
		1000	control_bit	rt	rs	01010			SLT(C)
		1000	control_bit	rt	rs	00110			SRL(C)
		1000	control_bit	rt	rs	00101			MUL(C)
		1000	control_bit	rt	rs	01011			SLL(C)
		1000	control_bit	rt	rs	11010			*SLLV(C)
		1000	control_bit	rt	rs	11011			*XOR(C)
		1000	control_bit	rt	rs	10110			*SRLV(C)
		1000	control_bit	rt	rs	10111			*SRAV(C)
		1000	control_bit	rt	rs	11111			*DIV(C)
	I-Type	1110	1	rt	imm[7:0]				LUI
		1100	1	rt	imm[7:0]				SLTI
		1101	1	rt	imm[7:0]				MULI
		0000	0	rt	imm[7:0]				BEQ
		0000	1	rt	imm[7:0]				BNE
		1111	1	rt	imm[7:0]				LW
		0011	1	rt	imm[7:0]				SW
		0001	0	rt	imm[7:0]				J
		0001	1	rt	imm[7:0]				JR
		1001	1	rt	imm[7:0]				JAL
		0101	1	rt	imm[7:0]				*SRA
		1011	1	rt	imm[7:0]				*ADDI

Table 3. Operation Control Values

Group Name	Operation	Operation Control			
MuSe Architecture	add	0	0	0	-
	sub	0	0	1	-
	and	0	1	0	-
	or	0	1	1	-
	mul	1	0	0	-
	sll	1	0	1	-
	srl	1	1	0	-
	slt	1	1	1	-
DoMe Architecture	add	0	0	0	0
	sub	0	0	0	1
	and	0	0	1	0
	or	0	0	1	1
	mul	0	1	0	0
	sll	0	1	0	1
	srl	0	1	1	0
	slt	0	1	1	1
	div	1	0	0	0
	xor	1	0	0	1

Table 4. MuSe Architecture Control Signal Values

Format	Instruction	RegDst	ALUSrc	RegWrite	MemRead	MemWrite	Branch	ALUOP	Jump	JumpReg	ShiftReg	Syscall
R	add	1	0	1	0	0	0	0	0	0	0	0
	sub	1	0	1	0	0	0	1	0	0	0	0
	mul	1	0	1	0	0	0	100	0	0	0	0
	and	1	0	1	0	0	0	10	0	0	0	0
	or	1	0	1	0	0	0	11	0	0	0	0
	sll	1	0	1	0	0	0	101	0	0	0	0
	srl	1	0	1	0	0	0	110	0	0	0	0
	jr	1	0	1	0	0	0	xxx	x	1	1	0
	syscall	0	0	0	0	0	0	xxx	x	0	0	1
	slt	1	0	1	0	0	0	100	0	0	0	0
I	lui	0	1	1	0	0	0	101	0	0	1	0
	slti	0	1	1	0	0	0	100	0	0	0	0
	muli	0	1	1	0	0	0	100	0	0	0	0
	beq	0	0	0	0	0	1	1	0	0	0	0
	bne	1	0	0	0	0	1	1	0	0	0	0
	sw	0	1	0	0	1	0	0	0	0	0	0
	lw	0	1	1	1	0	0	0	0	0	0	0
J	jal	0	0	1	0	0	0	xxx	1	0	0	0
	j	0	0	0	0	0	0	xxx	1	0	0	0

Table 5. DoMe Architecture Control Signal Values

Format	Instruction	ALUSrc	RegWrite	MemRead	MemWrite	ALUOp3	ALUOp2	ALUOp1	ALUOp0	Jump	Branch
**R**	add	0	1	0	0	0	0	0	0	0	0
	sub	0	1	0	0	0	0	0	1	0	0
	mul	0	1	0	0	0	1	0	0	0	0
	and	0	1	0	0	0	0	1	0	0	0
	or	0	1	0	0	0	0	1	1	0	0
	sll	0	1	0	0	0	1	0	1	0	0
	srl	0	1	0	0	0	1	1	0	0	0
	sllv	0	1	0	0	0	1	0	1	0	0
	srlv	0	1	0	0	0	1	1	0	0	0
	div	0	1	0	0	1	0	0	0	0	0
	xor	0	1	0	0	1	0	0	1	0	0
	srav	0	1	0	0	0	1	1	0	0	0
	slt	0	1	0	0	0	1	1	1	0	0
**I**	lui	1	1	0	0	0	1	0	1	0	0
	slti	1	1	0	0	0	1	0	0	0	0
	muli	1	1	0	0	0	1	0	0	0	0
	beq	0	0	x	0	0	0	0	1	0	1
	bne	0	0	x	0	0	0	0	1	0	1
	lw	1	1	1	0	0	0	0	0	0	0
	sw	1	0	0	1	0	0	0	0	0	0
	sra	1	1	0	0	0	1	1	0	0	0
	addi	1	1	0	0	0	0	0	0	0	0
	jr	x	0	x	0	x	x	x	x	1	x
	jal	x	1	x	0	x	x	x	x	1	x
	j	x	0	x	0	x	x	x	x	1	x

REFERENCES

MIPS (2001). Mips32 architecture for programmers. vol. ii: The mips32 instruction set.

Patterson, D. A. and Hennessy, J. L. (2016). *Computer organization and design ARM edition: the hardware software interface*. Morgan kaufmann.

Wallace, C. S. (1964). A suggestion for a fast multiplier. *IEEE Transactions on Electronic Computers*, EC-13(1):14–17.
